# A Fast and Robust UHPLC-MRM-MS Method to Characterize and Quantify Grape Skin Tannins after Chemical Depolymerization

**DOI:** 10.3390/molecules21101409

**Published:** 2016-10-21

**Authors:** Lucie Pinasseau, Arnaud Verbaere, Maryline Roques, Emmanuelle Meudec, Anna Vallverdú-Queralt, Nancy Terrier, Jean-Claude Boulet, Véronique Cheynier, Nicolas Sommerer

**Affiliations:** 1 Plate-Forme D’analyse des Polyphénols, UMR1083 Sciences Pour l’Œnologie, Institut National de la Recherche Agronomique, Montpellier 34060, France; pinassea@supagro.inra.fr (L.P.); verbaere@supagro.inra.fr (A.V.); maryline.roques@supagro.inra.fr (M.R.); meudec@supagro.inra.fr (E.M.); vallverd@supagro.inra.fr (A.V.-Q.); bouletjc@supagro.inra.fr (J.-C.B.); cheynier@supagro.inra.fr (V.C.); 2 Equipe Biosynthèse et Composition en Polyphénols et Polysaccharides, UMR1083 Sciences Pour l’Œnologie, Institut National de la Recherche Agronomique, Montpellier 34060, France; nancy.terrier@supagro.inra.fr; 3 Institut Français de la Vigne et du Vin, Pôle National Matériel Végétal, UMT Génovigne, Montpellier 34060, France

**Keywords:** grapes, tannins, phloroglucinolysis, quantitative UHPLC-QqQ-MS, method validation, metabolomics

## Abstract

A rapid, sensitive, and selective analysis method using ultra high performance liquid chromatography coupled with triple-quadrupole mass spectrometry (UHPLC-QqQ-MS) has been developed for the characterization and quantification of grape skin flavan-3-ols after acid-catalysed depolymerization in the presence of phloroglucinol (phloroglucinolysis). The compound detection being based on specific MS transitions in Multiple Reaction Monitoring (MRM) mode, this fast gradient robust method allows analysis of constitutive units of grape skin proanthocyanidins, including some present in trace amounts, in a single injection, with a throughput of 6 samples per hour. This method was applied to a set of 214 grape skin samples from 107 different red and white grape cultivars grown under two conditions in the vineyard, irrigated or non-irrigated. The results of triplicate analyses confirmed the robustness of the method, which was thus proven to be suitable for high-throughput and large-scale metabolomics studies. Moreover, these preliminary results suggest that analysis of tannin composition is relevant to investigate the genetic bases of grape response to drought.

## 1. Introduction

In the last few decades, climate change has become a reality and the effects have started to be perceived. Global warming, drought, and more drastic and frequent climate events affect nearly every crop and especially grapevines, which are highly sensitive to environmental conditions. Thus, it is anticipated that climate change, and especially drought, will increasingly impact grape development, affecting berry size, sugar content, acidity, phenolic composition, and consequently wine quality in areas where grapevines are traditionally cultivated [1,2].

Thus, it is necessary to explore grapevine response to climate change to better understand its genetic basis and assist with the selection of varieties with better adaptation potential.

Flavan-3-ols (including catechin monomers and proanthocyanidins, i.e., flavan-3-ol oligomers and polymers) are a complex family of plant flavonoids, naturally abundant in the grape skin. They are involved in plant response to biotic and abiotic stress and play an important role in the final quality of the wine as they influence its ageing potential, astringency, stability, and colour [3]. Grape skin proanthocyanidins are high molecular weight polymers, with average degrees of polymerisation (DP) around 30, and consist of catechin, epicatechin, gallocatechin, epigallocatechin, and epicatechin gallate units [4]. High variability in proanthocyanidin content and in the proportions of constitutive units and DPs has been reported both in natural populations and in segregating progenies [5]. Proanthocyanidin accumulation may be affected by environmental conditions, with a potential impact on plant response to stress and on the quality of plant-derived foods and beverages, including health benefits. For example, decreasing vine water status has led to increased proanthocyanidin levels in the skins of Syrah berries during berry growth [6] while no effect has been observed in other studies [7,8]. However, data on the impact of environment on berry proanthocyanidin composition are still limited. High throughput qualitative and quantitative methods are needed to evaluate proanthocyanidin content in programs aiming to investigate the genetic bases of plant response to environmental stress and identifying markers for selection of varieties with improved agronomic and quality characteristics.

**Figure 1:**
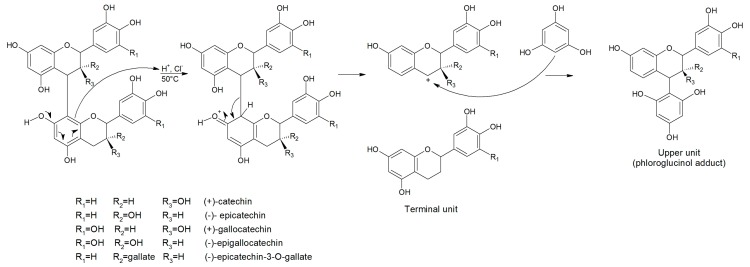
Acid-catalysed depolymerization of a proanthocyanidin dimer in the presence of phloroglucinol.

There are many methods to measure proanthocyanidins from natural products. Among them, methods based on HPLC analysis of proanthocyanidin units released after acid-catalysed depolymerisation in the presence of a nucleophilic agent are particularly useful, providing both quantitative and qualitative information, such as the nature and proportions of proanthocyanidin constitutive units and their average degree of polymerisation. These include phloroglucinolysis, using phloroglucinol as the nucleophilic agent, which has several advantages over methods using other common reagents such as unpleasant thiols [9]. After cleavage of B-type proanthocyanidin bonds, the terminal units are released as the corresponding free flavan-3-ol monomer and the upper units as intermediate carbocations that react with phloroglucinol to form stable adducts, as shown in Figure 1. Thus, grape skin tannins give rise to nine constitutive units, namely terminal units of catechin (C_term_), epicatechin (EC_term_), gallocatechin (GC_term_), epigallocatechin (EGC_term_) and epicatechin gallate (ECG_term_) and phloroglucinol adducts of upper units of catechin (C_up_), epicatechin (EC_up_), epigallocatechin (EGC_up_), and epicatechin gallate (ECG_up_).

Proanthocyanidin units can be detected by UV-visible spectroscopy (photo diode array, PDA) after HPLC separation. However, this method is not suitable for detection of terminal units of higher molecular weight proanthocyanidins which are present at very low concentration compared with the abundant upper units and are thus usually analysed using a more sensitive fluorimetric detector. Moreover, it requires a long elution gradient for separation of the different subunits as both PDA and fluorimetric detection lack specificity.

Ultra-high performance liquid chromatography (UHPLC) is a technique that allows faster chromatography and/or enhanced chromatographic resolution compared to classical HPLC, due to smaller bead size (1.7 µm vs. 3–5 µm) and increased column pressure (up to 1000 bar). UHPLC coupled with triple quadrupole mass spectrometry (UHPLC-QqQ-MS) using the multiple reaction monitoring (MRM) mode has been proposed as an alternative for fast analysis of phenolic compounds in grape and wine [10,11,12]. MRM detection is both very sensitive and highly specific because it is based on the analysis of parent and fragment ions that are characteristic of each target molecule. Thus, MRM functions can be overlapped, providing selectivity even on coeluted compounds under fast chromatography conditions, and this, combined with the large dynamic range of the QqQ mass spectrometer, allows a fast quantification method.

The objective of the present work was to develop a rapid and simple method based on UHPLC-MRM-MS to quantify major grape skin proanthocyanidin units after acid-catalysed depolymerisation in the presence of a nucleophilic agent (phloroglucinolysis).

## 2. Results and Discussion

### 2.1. Optimization

Several parameters had to be optimized, namely separation conditions and detection parameters. In order to reduce solvent consumption, as allowed by UHPLC with a solvent linear velocity much higher than with HPLC, we selected a 1 mm-diameter reversed-phase column. The flow rate was fixed to 0.17 mL∙min^−1^ with this 10 cm long column, which led to a column pressure between 600 and 950 bar during methanol elution. As already demonstrated in a previous work [12], the optimal percentage of formic acid in mobile phase for polyphenol analysis is 1%. The presence of acid improves chromatographic separation and ionization efficiency in the positive ion mode. Larger amount of acid leads to a signal decrease in ESI-MS. The column temperature was set to 40 °C in order to reduce methanol viscosity and thus to increase the mobile phase flow rate and linear velocity without degrading compounds. The chromatographic elution was adapted so as to obtain a short gradient without losing sensitivity in the MRM detection mode. This allows the elution of 9 compounds in a 6 min analysis. Including column washing and re-equilibration time, it was possible to have a cycle time of 10 min and inject 6 samples per hour.

**Figure 2:**
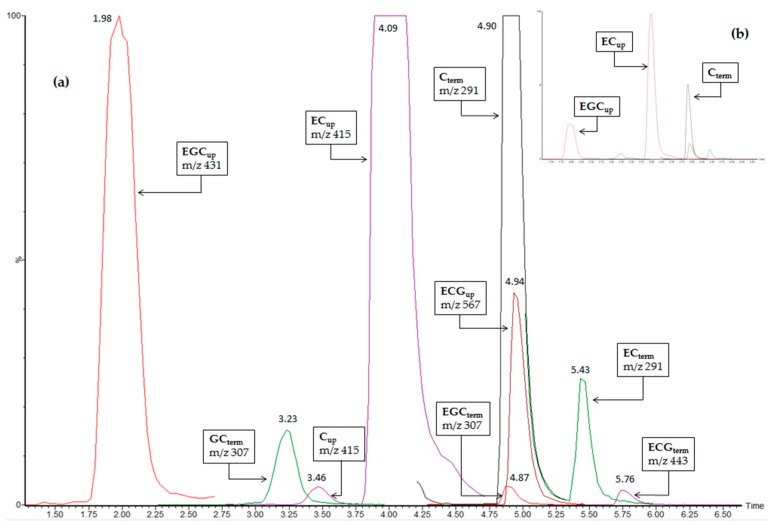
Superimposition of MRM traces from the UHPLC-QqQ-MS analysis of proanthocyanidin units of a grape skin extract after phloroglucinolysis; (**a**) 4× zoomed display; (**b**) full display.

The optimized chromatographic conditions are detailed in the Experimental section. The UHPLC-MRM-MS profiles obtained for all target compounds after injection of a grape skin extract after phloroglucinolysis are presented in Figure 2.

Direct infusion of standards in water/MeOH 50/50 (*v*/*v*) containing 1% formic acid provided the fragmentation pattern and collision energy suitable for an optimal detection of the 4 target analyte standards (C_term_, EC_term_, EGC_term_, ECG_term_). For the compounds that are not available as standards, additional experiments have been performed by injecting grape skin extracts after phloroglucinolysis to determine optimum ionization and fragmentation parameters. ESI provided singly charged positive [M + H]^+^ precursor ions for all compounds. Two MRM ion transitions were selected for each compound (Table 1); the most intense transition was used for quantification (quantifier ion), while the other one was used to increase selectivity and compound confidence (qualifier ion).

**Figure 3:**
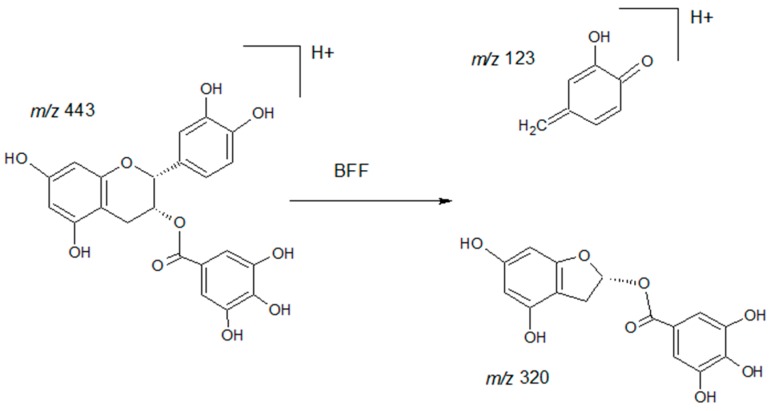
Fragmentation scheme for epicatechin gallate terminal unit in positive ionization mode. The fragmentation pathway involving benzofuran-forming fission (BFF) is demonstrated.

For terminal units of catechin, epicatechin, gallocatechin, and epigallocatechin, the main observed transition corresponds to the fragment ion at *m*/*z* 139 Th which results from a Retro-Diels-Alder (RDA) fragmentation of the C-ring. Identical fragments are obtained because only the neutral lost fragment differs between the different analytes. For epicatechin gallate terminal units, the obtained fragment at *m*/*z* 123 Th results from a benzofurane forming fission (BFF) which produces a C-ring benzofurane derivative (Figure 3) [13]. Upper units of catechin, epicatechin, and epigallocatechin were characterized by the fragment at *m*/*z* 127 Th which corresponds to the phloroglucinol. In the same way, identical fragments are obtained because only the neutral lost fragment differs between the different analytes. For the upper unit of epicatechin gallate, the fragment obtained was at *m*/*z* 153 Th, which corresponds to the galloyl which has lost a water molecule.

Flavan-3-ol monomers are commercially available, so each terminal unit was quantified with its standard except for gallocatechin which was quantified as equivalent of epigallocatechin (Table 1). For the phloroglucinol derivatives (C_up_, EC_up_, EGC_up_, and ECG_up_) which are not commercially available, the mass detector response was calibrated with standards from the same molecular family which is a suitable approach for methods developed for large-scale comparative studies. These compounds were quantified as equivalents of EC_term_ (Table 1).

A correction value was calculated for UHPLC-QqQ-MS detection to fit with the response of each compound determined after UHPLC-DAD analysis of the grape extract, with a longer gradient providing separation of all compounds (Supplementary Materials, Table S1). As demonstrated earlier [9] the response factors of upper and terminal units are almost the same in UV detection, so the correction values were calculated to have identical values with the two detection methods (calculations are provided as Supplementary Materials).

### 2.2. Method Validation

The performance of the proposed analytical method were thoroughly evaluated with a grape skin extract selected on the basis of its proanthocyanidin composition similar to the average composition of a panel sampling of skin extracts from 50 red and white grapes (Table 2). Concentration levels of the different units are highly heterogeneous, as already shown by Souquet et al. [4].

#### 2.2.1. Linearity

The linearity of the method was tested using standard solutions at ten concentration levels from 0.01 µmol/L to 333.33 µmol/L for catechin and epigallocatechin and from 0.02 µmol/L to 333.33 µmol/L for epicatechin and epicatechin gallate. The method yielded quadratic response for each compound. The weighted (1/*x*) calibration curves (*y* = a*x*² + b*x* + c) were found to be quadratic in the studied range with coefficient of determination (*R*^2^) above or equal 0.9961. The response obtained after dilution of the highest concentrations using linear calibration matched with those determined without dilution with the quadratic calibration curve.

#### 2.2.2. Recovery and Precision

Analyte recoveries were assessed by analyzing the percentage of recovery of each compound in grape extracts.

As pure proanthocyanidins are not commercially available, commercial extracts of white grape skins and of grape seeds, respectively containing 25% and 67% of proanthocyanidins (determined by UHPLC-DAD after phloroglucinolysis), were used.

Given the high heterogeneity of composition and to match with the concentration ranges of the calibration curve, two solutions were prepared: the first one is a 20 g/L solution of the white grape skin extract (1) and the second is a 20 g/L solution containing 50% of each extract (2). The grape seed extract provides catechin, epicatechin, and epicatechin gallate units while the white grape skin extract also provides epigallocatechin units.

The first solution was used to obtain two additional concentration levels of EGC_up_. For EGC_term_, a commercial standard solution (1 g/L, (3)) was used. Concentration ranges of the different units were obtained by mixing solutions (1), (2) and (3) in different proportions (A–G, Supplementary Materials, Table S2).

The grape skin extract (GE) solution was spiked with different volumes of solutions (1), (2), and (3) providing concentration levels A through G (Table 3). Concentration levels A and B were used only for EGC_up_ as explained above.

The lowest concentration levels were not applicable for all compounds as some concentration levels are lower than the variation coefficient of the mass signal.

The spiked samples were freshly prepared and analyzed over three consecutive days, with three replicates per day to evaluate interday and intraday precision. The percentage of recovery was calculated for each compound (9 analyses). 

Quantitative recoveries at all concentration levels tested ranged from 69.3% ± 3.8% to 132.7% ± 4.1%. Although for some units the concentrations in solution GE + C exceeded the highest concentration used for calibration (333 µmol/L), the recoveries of these compounds indicate that the calibration curve is still valid in this range.

Both intra- and inter-day precision studies presented satisfactory results for all compounds with RSDs below 6% and 9% (Table 3).

#### 2.2.3. Limits of Detection (LOD) and Quantification (LOQ)

The sensitivity of the method was evaluated by determining LODs and LOQs, corresponding to signal to noise ratios of 3, and 10, respectively (Table 3).

#### 2.2.4. Matrix Effects

Matrix effects result in suppression or enhancement of the signal [14]. They were evaluated by calculating the difference (in percentage) between the response recorded with the sample spiked with the solutions (1), (2) or (3) in different proportions (GE + A through GE + G, Table 3) and the response recorded with the same solutions diluted under the same conditions in solvent only.

Matrix-dependent enhancement/suppression effects were between 120.3% ± 6% and 85.0% ± 10% (Table 3).

#### 2.2.5. Stability

The stability of the processed sample was studied at 24 h and 48 h, largely exceeding sample storage time in the autosampler before injection.

No significant reduction of analyte concentration was observed after 48 h, except for the lowest concentration level of ECG_term_ (21%) and EC_term_ (15%). It is noteworthy that all compounds remained stable over 24 h for all concentration levels (Table 3).

Moreover, we checked the method for sensitivity stability over a 24 h period by regularly injecting a standard solution containing C, EC, EGC, ECG (0.01 mM each) between samples or sample batches. Grape extracts are crude matrices and a large number of samples were injected every day. Consequently, a 90 min daily cleaning of the system was necessary to maintain the system sensitivity. Critical points were on the UHPLC injection and tubing and on the mass spectrometer sampling cone. To minimize sample handling and injections, no dilution was carried out. Coupled with the daily cleaning of the system, this gave us appropriate repeatability and accuracy in a single injection, thus allowing an increased productivity.

### 2.3. Advantages of the Method

MRM is the method of choice to combine selectivity and sensitivity. Thanks to the selectivity of the MRM mode used for the detection, overlapped peaks are not synonymous with loss of information (Figure 2). Combined with the use of UHPLC rather than HPLC, it allows a very short analysis time: 10 min including column re-equilibration. Analysis time was optimized in order to be as short as possible. The 1.75 min before the first peak were necessary to keep a good separation, compulsory because of isomeric parent ion and fragment ion masses for C_up_ and EC_up_ and for C_term_ and EC_term_ (Figure 2), while the 4 min at the end of the elution program were necessary to flush the less polar compounds present in the extracts and re-equilibrate the column. Note that GC_up_, known to be present in very low amounts in grape skin proanthocyanidins and is coeluted with EGC_up_ under these conditions.

As shown in Figure 2, the high signal-to-noise ratio allowed for automated processing of data with simple optimized settings of the processing software. Predicted retention times and retention time windows were fixed in the processing method according to the values fixed in the acquisition method. With integration window extents fixed to 2 min, no integration correction had to be made, thus allowing fast, automatic and more robust data treatment.

**Figure 4:**
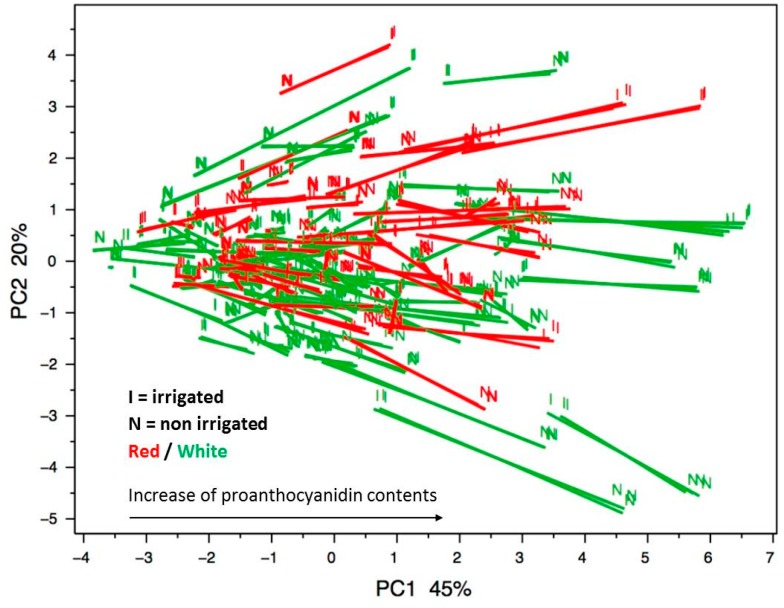
Projection of the grape samples on the first two principal components of the PCA performed on the skin flavan-3-ol composition data (C_up_, EC_up_, EGC_up_, ECG_up_, C_term_, EC_term_, GC_term_, EGC_term_ and ECG_term_; mg/g berry skin; centred reduced data).

Triplicate analysis of a series of 107 grape skin extracts confirmed the high robustness of the method (Table 3, intraday precision). This is illustrated by the proximity of the projections of triplicate samples on the first two axes of the principal component analysis performed on the proanthocyanidin composition data (Figure 4). These results indicate that only one analytical injection is necessary, which is saves a great deal of time and solvent for large-scale studies. In addition, the very large dynamic range of the method allows, in a single injection, quantification of compounds for which the variation between the smallest and the highest concentration is up to 10^4^ (Table 2).

The sensitivity of the method allows a simple and fast sample preparation with no prior purification. About 20 min are needed to carry out the phloroglucinolysis reaction and obtain a suitable sample for injection. For large-scale studies, about 60 samples per day can be analysed including standard calibration, quality controls and blanks.

### 2.4. Application to Large-Scale Analysis of Grape Skin Proanthocyanidin Composition

The applicability of the method was demonstrated through a large-scale comparative study using grape berries collected from 107 different grape cultivars grown under two conditions in the vineyard, irrigated and non-irrigated.

**Figure 5:**
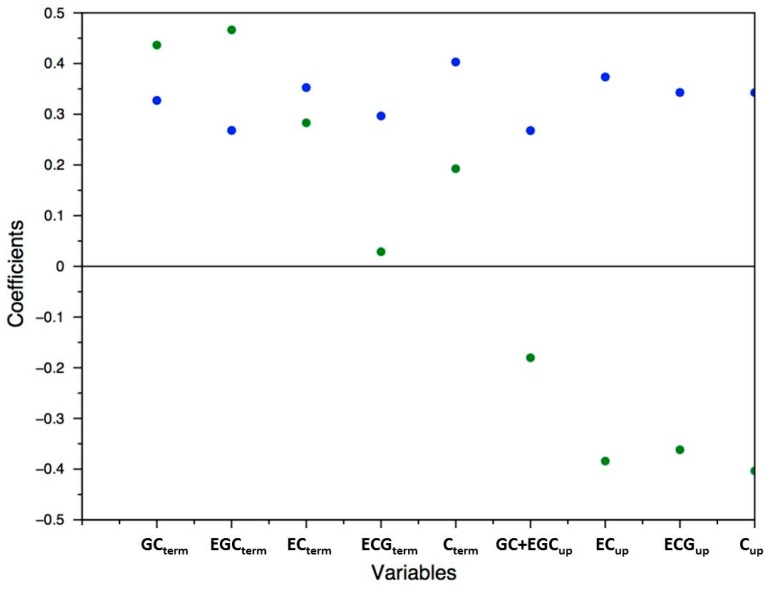
Projection of the variables on the first two principal components, PC1 (●) and PC2 (●), of the PCA performed on the skin flavan-3-ol composition data mg/g berry skin; centered reduced data).

A principal component analysis (PCA) was performed on the proanthocyanidin composition data of 214 grape skin samples (107 varieties grown under irrigated and non-irrigated conditions) analyzed in triplicate. Projection of the samples on the first two principal components, explaining 65% of the variability, illustrates the variability of proanthocyanidin skin composition in the collection (Figure 4). Projection of variables (concentration of the 9 proanthocyanidin constitutive units in mg/g berry skin) on the first two principal components is shown in Figure 5. The first axis was positively associated to all proanthocyanidin units. The second axis, accounting for 20% of the variability, contrasted terminal units with upper units. Red and white varieties appear evenly distributed in the chemical space, indicating that tannins are as abundant in white grapes as in red grapes and that skin tannin composition is not related to grape color. Comparison of the irrigated/non-irrigated samples is also illustrated, highlighting an influence of irrigation on tannin content. Thus, most samples are shifted negatively along the first axis upon irrigation, indicating a decrease of tannin contents in the skins of irrigated grapes. Some samples are shifted in the opposite direction, indicating differences in the cultivar responses to drought. Shifts along the second axis reflected qualitative modifications, mostly irrigated grapes containing larger concentrations of terminal units and lower amounts of upper units and thus tannins with lower DPs than the non-irrigated samples.

## 3. Experimental Section

### 3.1. Chemicals

Formic acid and HPLC grade methanol were purchased from Sigma Aldrich (St Louis, MO, USA). Deionized water was obtained from a Milli-Q purification system (Millipore, Molsheim, France). Standards of catechin, epicatechin and epicatechingallate were purchased from Sigma-Aldrich. Standard of epigallocatechin was purchased from Extrasynthese (Geney, France). Acetone, hydrochloric acid, trifluoroacetic acid, phloroglucinol, l-ascorbic acid, and ammonium formiate were purchased from Sigma Aldrich.

### 3.2. Samples

Samples were collected from an association panel of 214 *Vitis vinifera* cultivars designed from the French National Grapevine Germplasm Collection (Domaine de Vassal, INRA, Marseillan, France) grown under two conditions in the vineyard, irrigated and non-irrigated [15].

### 3.3. Standard and Sample Preparation

Standard solutions were prepared in methanol/H_2_O 50/50 (*v*/*v*) 1% formic acid. Their calibration ranges are summarized in Table 1.

#### 3.3.1. Sample Preparation for UHPLC-QqQ-MS Analysis

Sample preparation was adapted from Kennedy et al. [7]. Ratio of grape powder mass and solvent volume was optimized on grapes showing high proanthocyanidin skin contents, selected from Huang et al. [3] data. Skins were isolated from the grapes and milled with liquid nitrogen with a Mortar Grinder Pulverisette 2 (Fritsch, Idar-Oberstein, Germany). 100 mg of powder were weighed and 500 µL of methanol were immediately added. Then 3.5 mL of acetone/H_2_O 70/30 (*v*/*v*) 0.05% trifluoroacetic acid were added. The mixture was crushed with Precellys (Bertin Technologies, Montigny-le-Bretonneux, France) during three cycles (3 × 40 s each). After centrifugation (4500 rpm, 5 min, 4 °C), 1 mL of the supernatant was taken to dryness under vacuum with Genevac (SP Scientific, Warminster, PA, USA).

#### 3.3.2. Sample Preparation for Method Validation

The grape skin extract (grape variety Roublot) chosen for method validation (Table 2) was prepared as described in 3.3.1. Solution (1) (white grape skins 20 g/L) was prepared as follows: exactly 100 mg of white grape skin extract were weighed and diluted with 5 mL of methanol/H_2_O 50/50 (*v*/*v*). Solution (2) (50/50 grape skin/seed extracts 20 g/L) was prepared as follows: exactly 0.5 g of each extract were weighed and diluted with 50 mL of methanol/H_2_O 50/50 (*v*/*v*).Extracts used to prepare solutions (1) and (2) were provided by Grap’Sud (Cruviers-Lascours, France). Solution (3) (commercial standard of epigallocatechin 1 g/L) was prepared as follows: exactly 5 mg were weighed and diluted with 5 mL of methanol/H_2_O 50/50 (*v*/*v*). Then, solids obtained after evaporation with Genevac (SP Scientific, Warminster, PA, USA) were spiked with different volumes of solutions (1), (2) and (3), as described in Table 3. Mixtures obtained were dried again with Genevac.

#### 3.3.3. Phloroglucinolysis Reaction

The phloroglucinolysis reaction was carried out on the solid obtained after evaporation with Genevac. After optimization, 700 µL of phloroglucinol/ascorbic acid solution (respectively 50 and 10 g/L in MeOH/HCl 0.2 M) were added. After solubilisation with an Ultrasonic bath (VWR, Fontenay-sous-Bois, France) (30 min), the solution obtained was heated in a water bath (Julabo ED, Seelbach, Germany) (50 °C, 20 min). The phloroglucinolysis reaction was stopped by placing the sample in ice and by adding 700 µL of ammonium formiate solution (12.6 g/L). The solution obtained was centrifuged (HettichLab Technology, Tuttlingen, Germany) (15,000 rpm, 10 min, 4 °C). The supernatant was filtered through a 0.2 µm RC Membrane filter (Phenomenex, Le Pecq, France) before injection.

### 3.4. UHPLC-QqQ-MS Conditions

UHPLC-MRM-MS analyses were carried out using an Acquity UPLC system (Waters, Saint-Quentin-en-Yvelines, France) hyphenated to a triple quadrupole (QqQ) TQD mass spectrometer (Waters, Saint-Quentin-en-Yvelines, France) operating in MRM mode with electrospray ionization (ESI) in positive ion mode. The diode array detection (DAD) spectra were recorded in the range of 210−600 nm (resolution 1.2 nm). The UPLC system included a binary pump, a cooled autosampler maintained at 7 °C and equipped with a 5-μL sample loop, a 100-μL syringe and a 30-μL needle, and a DAD. MassLynx software was used to control the instruments and to acquire the data and TargetLynx software was used to process the data. The column used for chromatographic separation was a reversed-phase Acquity HSS T3 1.8 μm 1.0 mm × 100 mm (Waters, Saint-Quentin-en-Yvelines, France) protected by a 0.2 μm in-line filter and maintained at 40 °C. The mobile phase consisted of 1% (*v*/*v*) formic acid in Milli-Q water (solvent A) and 1% (*v*/*v*) formic acid in methanol (solvent B). The elution program was as follows: isocratic for 1.5 min with 2% B, 2%−7% B (1.5 min), 7%−40% B (2 min), 40%−99% B (1 min), isocratic for 0.5 min with 99% B, 99%−2% B (0.5 min) followed by washing and reconditioning of the column (3 min). The source and desolvation temperatures were respectively set at 120 and 450 °C. Nitrogen was used as desolvation (500 L/h) and cone (50 L/h) gas. Argon was used as collision gas at a flow rate of 0.16 mL/min. Capillary voltage was set at 3.5 kV in positive. Samples were filtered through a 0.22 μm PTFE filter (Waters) and injected into the column by using the Partial Loop with Needle Overfill injection mode with an injection volume of 1 μL. The flow rate was 0.170 mL/min and the oven temperature was maintained at 40 °C.

### 3.5. Chemometrics

Standardized PCA was performed with Scilab (www.scilab.org) using the Fact toolbox. The data contained 9 variables and 642 observations: 107 *V. vinifera* varieties, irrigated or not, and 3 analytical repetitions.

## 4. Conclusions

We have developed a fast UHPLC-QqQ-MS analysis method (10 min, single injection) for the quantification of the nine residues obtained after tannin depolymerisation by phloroglucinolysis. Our method combines selectivity and sensitivity enhanced by the use of UHPLC to shorten chromatographic elution and increase peak height. The MRM detection allows for a robust analytical throughput of 60 samples per 24 h without the need for duplicate injections, which saves considerable time compared to less robust methods. Even though grape extracts are crude matrices, no sample dilution was necessary; only a limited daily cleaning of the system is required to avoid a sensitivity loss. The optimized automatic data treatments allow simple, robust, and fast data processing, independent of operator decisions or know-how. The method was validated and shows satisfactory data for all parameters tested.

The proposed method has been successfully applied to a large-scale comparative study in order to better characterize the grapevine response to drought.

## Figures and Tables

**Table 1:** List of quantified compounds, MRM parameters (ion mode, precursor and product ions *m*/*z*, retention times), and calibration ranges.

Position	Compound	Ion Mode	*m*/*z* Precursor Ion ^a^ (Th)	*m*/*z* Quantifier ^a^ (Th)	*m*/*z* Qualifier ^a^ (Th)	Retention Time (min)	Calibration Range (µmol/L)
Upper units	C_up_	+	415.1	127.1	289.1	3.46	as equivalents of EC_term_
	EC_up_	+	415.1	127.1	289.1	4.09	as equivalents of EC_term_
	EGC_up_	+	431.2	127.1	305.1	1.98	as equivalents of EC_term_
	ECG_up_	+	567.2	153.1	247.2	4.94	as equivalents of EC_term_
Terminal units	C_term_	+	291.1	139.0	123.1	4.90	0.01–333.33 µmol/L
	EC_term_	+	291.1	139.0	123.1	5.43	0.02–333.33 µmol/L
	GC_term_	+	307.1	139.0	151.0	3.23	as equivalents of EGC_term_
	EGC_term_	+	307.1	139.0	151.0	4.87	0.01–333.33 µmol/L
	ECG_term_	+	443.1	123.1	273.1	5.76	0.02–333.33 µmol/L

^a^ span: 0.2. +, positive.

**Table 2:** Composition of the grape skin extract used for validation compared to the range of concentrations and average concentrations measured in a panel of 50 red and white grape skin extracts.

Grape Skin Extract	C_up_	EC_up_	EGC_up_	ECG_up_	C_term_	EC_term_	GC_term_	EGC_term_	ECG_term_
	Concentration (µmol/L)								
Composition of the extract used for validation	0.6	168.9	104.6	14.2	13.3	0.9	4.0	0.7	0.4
Average of measured concentrations	0.6	109.5	51.0	7.1	14.1	1.3	4.7	0.7	0.4
Lowest measured concentration	0.14	18.0	1.5	0.9	3.1	0.2	0.4	0.02	0.06
Highest measured concentration	1.6	270.1	131.3	31.2	48.4	4.5	11.5	3.7	1.9

**Table 3:** Concentration levels (µmol/L) for each compound in the solutions used for method validation and method validation parameters: limits of detection (LOD) and quantification (LOQ), recovery and precisions intra and inter-day, suppression/enhancement effect and stability at different concentration levels (GE: grape skin extract used for method validation (Table 2) A–G: standard solutions, Supplementary Materials, Table S2).

	EC_up_	EGC_up_	ECG_up_	C_term_	EC_term_	EGC_term_	ECG_term_
	Concentration (µmol/L)						
GE + A		238.1					
GE + B		166.6					
GE + C	1074	171.7	101.4	536.7	489.2		110
GE + D	358.4	98.9	33.4	141.3	122.8	47.4	27.7
GE + E	179.6	80.7	15.9	17.7	8.3	3.4	2
GE + F	131.9	75.9	11.2	9.8	0.9	0.5	0.4
GE + G	121.8	74.8	10.3	9.5	0.6		0.3
GE	120	74.3	10	9.5	0.643	0.5	0.285
LOD (µmol/L)	0.026	0.008	0.029	0.021	0.008	0.002	0.007
(nmol/g of grape skin)	1.456	0.448	1.624	1.176	0.448	0.112	0.392
LOQ (µmol/L)	0.086	0.027	0.096	0.070	0.026	0.006	0.023
(nmol/g of grape skin)	4.816	1.512	5.376	3.920	1.456	0.336	1.288
	**Recovery (%)**						
A	n.d	95.0 ± 5.1	n.d	n.d	n.d	n.d	n.d
B	n.d	101.0 ± 5.9	n.d	n.d	n.d	n.d	n.d
C	98.0 ± 4.1	92.3 ± 5.4	85.0 ± 5.6	106.7 ± 4.6	132.7 ± 4.1	n.a	110.7 ± 4.9
D	104.3 ± 2.5	98.7 ± 3.4	98.3 ± 4.4	97.3 ± 3.5	124.3 ± 1.9	70.7 ± 2.8	114.0 ± 2.1
E	112.7 ± 2.9	n.a	95.0 ± 3.4	102.0 ± 4.4	127.3 ± 2.9	69.3 ± 3.8	113.3 ± 2.1
F	n.a	n.a	n.a	103.4 ± 0.6	116.3 ± 1.7	73.3 ± 2.0	107.0 ± 1.8
G	n.a	n.a	n.a	n.a	118.0 ± 3.3	n.a	108.3 ± 3.1
	**Interday Precision (RSD %)**						
C	5	9	5	6	7	n.a	7
D	3	6	6	3	3	3	5
E	6	n.a	6	5	6	3	4
F	n.a	n.a	n.a	5	5	3	7
G	n.a	n.a	n.a	n.a	8	n.a	9
	**Intraday Precision (RSD %)**						
A	n.d	5	n.d	n.d	n.d	n.d	n.d
B	n.d	6	n.d	n.d	n.d	n.d	n.d
C	4	5	5	5	6	n.a	6
D	2	3	4	3	2	2	2
E	3	n.a	3	5	5	3	2
F	n.a	n.a	n.a	1	2	1	2
G	n.a	n.a	n.a	n.a	3	n.a	3
	**Suppression/Enhancement Effect (%)**						
A	n.d	85.0 ± 10.0	n.d	n.d	n.d	n.d	n.d
B	n.d	96.0 ± 13.0	n.d	n.d	n.d	n.d	n.d
C	99.7 ± 4.3	105.0 ± 28.3	91.3 ± 5.3	104.0 ± 5.0	103.0 ± 4.0	n.a	97.7 ± 5.0
D	105.0 ± 2.3	91.0 ± 21.7	92.0 ± 4.6	104.3 ± 3.6	103.7 ± 2.0	110.3 ± 3.0	99.7 ± 2.0
E	120.3 ± 6.0	n.a	95.6 ± 5.0	110.3 ± 5.0	107.0 ± 4.3	106.7 ± 4.3	104.7 ± 2.3
F	n.a	n.a	n.a	116.3 ± 1.0	99.3 ± 2.0	101.3 ± 2.3	94.7 ± 1.7
G	n.a	n.a	n.a	n.a	99.3 ± 3.3	n.a	100.0 ± 3.7
	**Stability (% loss)**						
24 h							
C	8	8	1	7	n.a	n.a	8
E	4	n.a	5	4	1	1	4
G	n.a	n.a	n.a	n.a	7	n.a	3
48 h							
C	9	6	0	8	8	n.a	9
E	7	n.a	2	4	2	4	10
G	n.a	n.a	n.a	n.a	15	n.a	21

n.d: non detected; n.a: not applicable (value lower than the variation coefficient of the mass signal).

## Supplementary Materials

Supplementary materials can be accessed at: http://www.mdpi.com/1420-3049/21/10/1409/s1.
