# Supplementary material for: A Fast and Robust UHPLC-MRM-MS Method to Characterize and Quantify Grape Skin Tannins after Chemical Depolymerization
Source: Molecules. 2016 Oct 21;21(10):1409. doi: 10.3390/molecules21101409 (PMC6273201; doi:10.3390/molecules21101409)
Supplement: Supplementary file 1 [file molecules-21-01409-s001.pdf]

Supplementary Materials: A Fast and Robust UHPLC-MRM-MS Method to Characterize and Quantify Grape Skin Tannins after Chemical Depolymerization

Lucie Pinasseau, Arnaud Verbaere, Maryline Roques, Emmanuelle Meudec, Anna Vallverdú-Queralt, Nancy Terrier, Jean-Claude Boulet, Véronique Cheynier and Nicolas Sommerer

Table S1. Correction values

	Correction Value
Catechin upper unit	2.8
Epicatechin upper unit	2.8
Epigallocatechin upper unit	11.6
Epicatechin gallate upper unit	2.4

Calculation of correction values

With the UV detection, the molar ratio (R) between EC_{term} and the upper unit (X_{up}) is calculated:

$$R = \frac{\text{ECterm}}{\text{Xup}} \rightarrow \text{UV detection} \qquad \text{(S1)}$$

With the MRM detection, the same ratio (R) has to be obtained. So, the theoretical area of the upper unit is calculated (area $Xup_{theo)}$ compared to the real obtained area of EC_{term} in order to have the same ratio (R) than in UV:

$$\text{Area } Xup_{theo} = \frac{\text{area ECterm}}{\text{R}} \rightarrow \text{MRM detection} \qquad \text{(S2)}$$

Then, the correction value to be applied to the upper unit (correction value X_{up}) corresponds to the ratio between the theoretical area of the upper unit (area Xup_{theo}) and the experimental area obtained with MRM detection of the upper unit (area Xup_{exp}):

$$\text{Correction value x up} = \frac{\text{area Xuptheo}}{\text{area Xupexp}} \rightarrow \text{MRM detection} \qquad \text{(S3)}$$

Table S2. Concentration levels (µmol/L) for each compound in the standard solutions used for method validation.

	**EC_{up}**	**EGC_{up}**	**ECG_{up}**	**C_{term}**	**EC_{term}**	**EGC_{term}**	**ECG_{term}**
	**(1) Solution of White Grape Skins at 20 g/L**						
A (400 µL of (1))	n.d	572.0	n.d	n.d	n.d	n.d	n.d
B (300 µL of (1))	n.d	429.0	n.d	n.d	n.d	n.d	n.d
	**(2) Solution of 50/50 Grape Skins/Seeds at 20 g/L and (3) Commercial Standard Solution of EGC at 1 g/L**						
C (400 µL of (2))	3339.2	339.3	320.8	1845.1	1709.8	n.a	384.0
D (200 µL of (2) + 164 µL of (3))	1669.6	169.6	160.4	922.5	854.9	400.0	192.0
E (50 µL of (2) + 41 µL of (3))	417.4	n.a	40.1	230.6	213.7	100.0	48.0
F (10 µL of (2) + 2µL of (3))	n.a	n.a	n.a	46.1	42.7	5.0	9.6
G (1 µL of (2))	n.a	n.a	n.a	n.a	4.3	n.a	1.0

n.d: non detected; n.a: not applicable.
